# Clusterin and pentraxin 3 are markers of severity during febrile neutropenia in adults with haematological malignancies receiving intensive chemotherapy

**DOI:** 10.1111/bjh.70002

**Published:** 2025-07-28

**Authors:** Coralie Mallebranche, Carole Mosnier, Corentin Orvain, Jérémie Riou, Sylvain Thepot, Pascale Pignon, Simon Blanchard, Nathalie Tortevoie, Isabelle Pellier, Mathilde Hunault‐Berger, Pascale Jeannin, Yves Delneste, Aline Schmidt‐Tanguy, Céline Beauvillain

**Affiliations:** ^1^ Université Angers, Université de Nantes, CHU Angers, Inserm, CNRS, CRCI2NA, SFR ICAT Angers France; ^2^ Unité d'onco‐Immuno‐hémato pédiatrique, CHU d'Angers Angers France; ^3^ Service Des Maladies du Sang CHU Angers Angers France; ^4^ Fédération Hospitalo‐Universitaire ‘Grand‐Ouest Acute Leukemia’ (FHU‐GOAL) Angers France; ^5^ Département de Biostatistiques et de Méthodologie, CHU Angers Angers France; ^6^ Laboratoire d'Immunologie et Allergologie, CHU d'Angers Angers France

**Keywords:** infection, leukemia, neutropenia, sepsis

## Abstract

The identification of predictive biomarkers of severity can improve patient management because febrile neutropenia can be associated with significant morbidity and mortality. We prospectively studied pentraxin 3 (PTX3), a soluble innate immunity receptor, and clusterin (CLU), a chaperone protein that binds extracellular histones during sepsis, in patients experiencing febrile neutropenia after intensive treatment for haematological malignancies. One hundred and forty‐three adult patients were included, with 158 episodes of neutropenia analysed. Febrile neutropenia was observed in 119 patients (75%), including 62 without sepsis (group 1; 53%), 42 with sepsis (group 2; 35%) and 14 with severe sepsis/septic shock (group 3; 12%) (missing data, MD): (1) Among these patients, 94 patients had a quick Sequencial Organ Failure Assessment score (qSOFA) <2 (84%) and 18 had qSOFA ≥2 (16%) (MD: 7). PTX3 levels increased significantly more in group 3 than in group 1 (*p* = 0.009). CLU levels tended to decrease after fever more in patients with qSOFA ≥2 than in those with qSOFA <2 and more in patients in group 3 than in group 1 but without a significant difference. PTX3 and CLU could be biomarkers of severity in patients with febrile neutropenia in adults. Their use to tailor the initial management of febrile neutropenia should be prospectively evaluated.

## Graphical abstract

During febrile neutropenia in patients with haematological malignancies, pentraxin 3 (PTX3), a soluble innate immunity receptor, increases with severe sepsis, while clusterin, a chaperon protein involved in extracellular histones clearance, decreases in patients with quick SOFA score (qSOFA) ≥2.

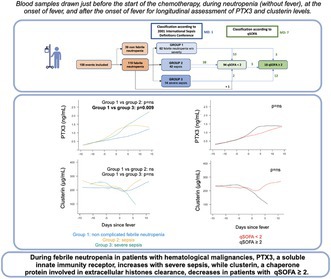

**Abbreviations**
ALL: acute lymphoid leukaemia
AML: acute myeloid leukaemia
CLU: clusterin
CRP: C‐reactive protein
DAMP: damage‐associated molecular patterns
FN: febrile neutropenia
HCT: haematopoietic cell transplantation
PRR: pattern recognition receptors
PTX3: pentraxin 3

## INTRODUCTION

Febrile neutropenia (FN) is a frequent complication in patients with haematological malignancies receiving intensive chemotherapy treatments and is often due to bacterial or fungal infections that can cause sepsis or septic shock.^1^, ^2^ The risk of mortality after septic shock in these patients reaches 40%–50%.^3^, ^4^ Although the use of empirical antibiotics greatly reduces mortality, it remains around 10%.^1^ Few specific biomarkers are available to quickly predict the type of pathogen and the severity of FN and to adapt patient care. The most widely studied and validated are C‐reactive protein (CRP) and procalcitonin.^5^

Pattern recognition receptors (PRR) are a diverse group of cellular or soluble sensors belonging to the innate immune system. Soluble PRR include, among others, complement components, surfactant proteins, mannose binding lectin, ficolins and pentraxins.^6^ The roles of PRR are to recognize pathogen‐associated molecular patterns and damage‐associated molecular patterns (DAMP); activate complement; promote phagocytosis through opsonization; and regulate inflammation.^6^ In addition to CRP, PRR include pentraxin 3 (PTX3), a long pentraxin produced by endothelial and phagocytic cells such as neutrophils.^7^, ^8^ Both CRP and PTX3 levels increase during infection, particularly bacterial infections, and may be useful in predicting sepsis.^9^, ^10^, ^11^, ^12^ Since PTX3 is released earlier than CRP during inflammation, PTX3 is an attractive biomarker for patients with neutropenia that require prompt and appropriate care.^13^, ^14^, ^15^

Clusterin (CLU) is a multifunctional protein with multiple properties that act as an apolipoprotein, a complement regulator and an extracellular chaperone.^16^ As a chaperone, CLU stabilizes a wide range of proteins and prevents the formation of toxic aggregates,^17^ thereby promoting their clearance. These properties extend to toxic proteins, such as certain DAMP, giving CLU a role in tissue protection by limiting apoptosis and necrosis, attenuating inflammation and reducing autoimmune responses.^18^, ^19^, ^20^ Recently, we demonstrated that CLU can bind to extracellular histones and neutralizes their cytotoxic effects.^21^ Following severe organ injury resulting in cell death or during NETosis, histones can be released, causing endothelial damage and promoting inflammatory response and thrombosis. During sepsis, serum CLU levels decrease, with a prolonged decrease associated with mortality.

In FN, biomarkers are needed to evaluate the severity of sepsis during bacterial infections, as well as in other types of infection (viral or fungal). These biomarkers should reflect tissue injury, enabling the selection of the most appropriate treatment or medical unit for the patient. No previous study has explored the variation of CLU in neutropenic patients. The aim of our study is to describe and evaluate the variations in PTX3 and CLU levels during chemotherapy‐induced FN, with a particular focus on assessing whether these soluble innate proteins could be valuable biomarkers.

## PATIENTS AND METHODS

### Study cohort and ethical considerations

This prospective study included adult patients aged ≥18 years hospitalized at Angers University Hospital between March 2014 and October 2017 for acute lymphoid leukaemia (ALL) induction, acute myeloid leukaemia (AML) induction or consolidation or high‐dose chemotherapy with autologous haematopoietic cell transplantation (HCT) as consolidation treatment for multiple myeloma or lymphoma. Patients with acute promyelocytic leukaemia were not included. The same patient may be included several times during successive courses of chemotherapy. This study was conducted in accordance with the Declaration of Helsinki and its later amendments. Written informed consent was obtained from all patients.

### Definitions

Neutropenia is defined as a neutrophil count ≤0.5 G/L. Fever is defined as a body temperature ≥38.3°C once or ≥38°C at least twice within 1 h.^22^

Patient data were classified according to two different classifications: recommendations from the 2001 International Sepsis Definitions Conference^23^ according to the severity of the infectious event and according to the quick Sequential Organ Failure Assessment score (qSOFA) at the onset of fever.^24^ The first classification divided into three ‘fever groups’: ‘on‐complicated FN—without sepsis’ (group 1), including patients with FN and systemic inflammatory response syndrome (SIRS); ‘Sepsis’ (group 2); and ‘Severe sepsis’ (group 3) including patients with severe sepsis or septic shock. Sepsis was defined as SIRS with a documented or suspected infection, severe sepsis as sepsis with at least one organ injury and septic shock as severe sepsis with ongoing arterial hypotension despite adequate fluid resuscitation and requiring the use of vasopressor therapy. Concerning qSOFA, the presence of at least two criteria was considered a poor prognostic factor, and patients were classified into two groups: qSOFA <2 and qSOFA ≥2.

### Biological analyses

Blood samples were drawn just before the start of the chemotherapy, during neutropenia (without fever), at the onset of fever (designated as day 0 or D0) and 48 h after the onset of fever (day 2 or D2). Serum samples were frozen at −80°C until use. PTX3 levels were evaluated using a homemade ELISA assay, including an anti‐human PTX3 monoclonal antibody (MNB4 clone) as capture Ab (provided by Mantovani's lab) and a polyclonal PTX3 antibody as detection antibody.^8^, ^25^ Human CLU was quantified on serum using a commercial ELISA kit (Human CLU Quantikine R&D® Systems, Minneapolis, MN, USA), according to the manufacturer's recommendations. IL‐6, IL‐8, TNFα and IL‐1β were quantified using a magnetic Luminex® assay (Bioplex200®, BioRad, Hercules, CA, USA), according to the manufacturer's recommendations.

### Statistical analysis

Categorical and continuous variables were compared by *χ* ^2^ or Fisher exact tests and by the Mann–Whitney test respectively. Multiple comparison analyses used the Kruskal–Wallis test. To study associations between qSOFA or Fever Group variables and the trajectory of biomarkers, a linear mixed model was computed. The fixed effects chosen for the analyses were time and time^2^ to capture the parabolic trajectories of biomarkers of interest. We also added a random intercept to the model to capture inter‐ and intra‐individual variability. Statistical analyses were performed with GraphPad Prism (version 10.1) and R software (version 3.6.1).^26^

## RESULTS

### Characteristics of patients and of episodes of FN

**FIGURE 1:**
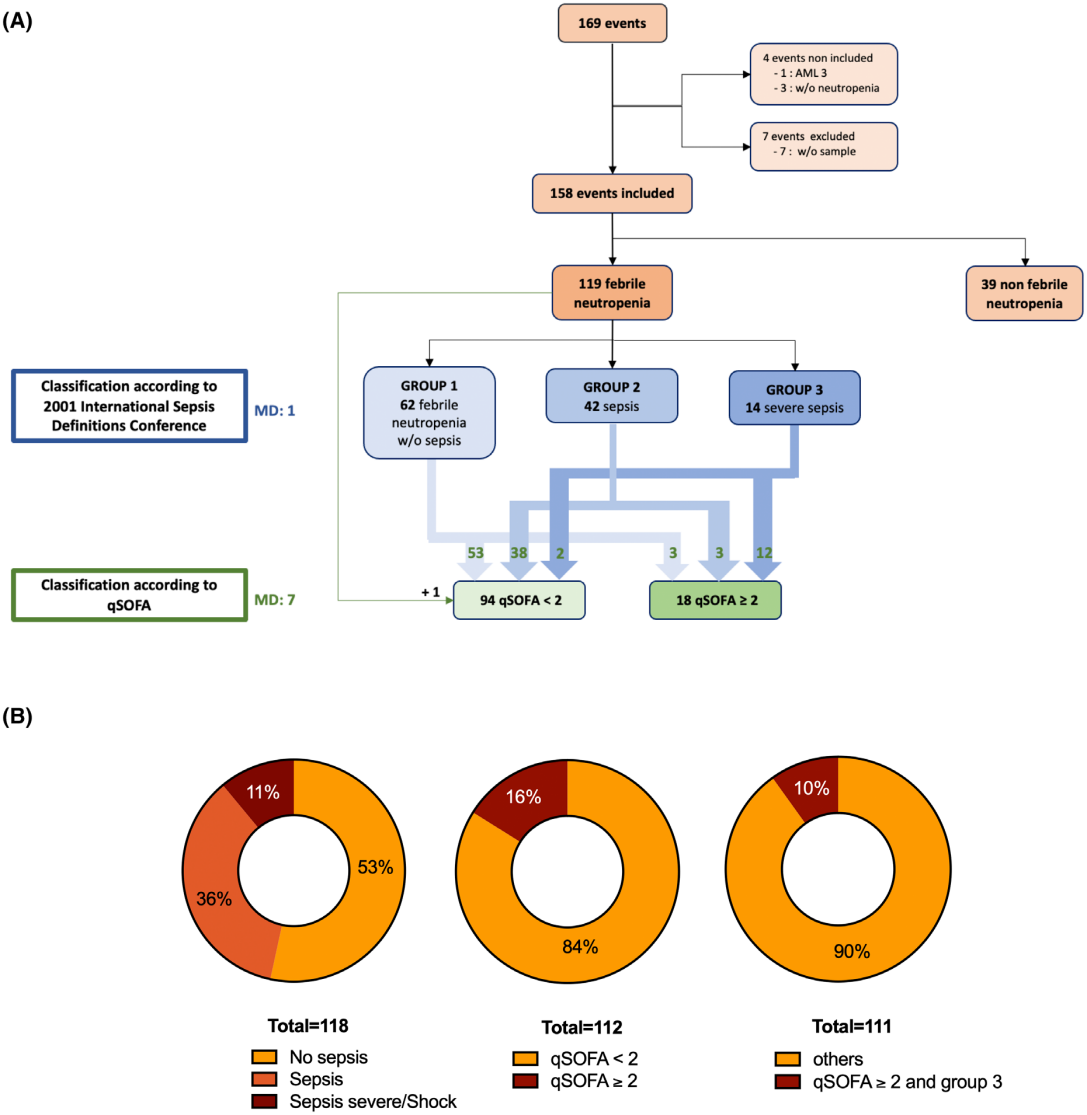
(A) Flow chart illustrating the process of data analysis in the study. (B) Distribution of patients by classification. Numbers in each square indicate the number of participants at each step. For six patients from group 1 and one patient from group 2, data were not available for qSOFA classification. AML, acute myeloid leukaemia; MD, missing data; qSOFA, quick SOFA score; w/o, without.

One hundred and forty‐two patients were screened between March 2014 and October 2017 for a total of 169 events evaluated, of which 158 were included and analysed (Figure 1). Fourteen patients were included twice during different courses and one patient was included three times. Median age at diagnosis was 55 years [min–max: 18–74]. Median duration of neutropenia was 9 days [min–max: 2–68]. One hundred and nineteen cases (75%) progressed to FN. FN was more frequent during treatment for lymphoma (*p* = 0.006) and less frequent during treatment of ALL (*p* < 10^−4^) (Table 1).

**TABLE 1:** Patient characteristics by febrile neutropenia occurrence.

Clinical characteristics***	Non‐febrile neutropenia	Febrile neutropenia	Total	*p*‐value
	39 (25)	119 (75)	158	
Sex, *n* (%)				
Male	24 (62)	72 (61)	96 (61)	0.99**
Female	15 (38)	47 (39)	62 (39)	
Median age at diagnosis (years) [Min–Max]	54 [20; 74]	56 [18; 71]	55	0.37***
Type of disease and treatment, *n* (%)				**<10** ^−4^*
AML	14 (36)	49 (41)	63 (40)	0.58**
ALL	11 (28)	5 (4)	16 (10)	**<10** ^−4^**
Lymphoma	4 (10)	38 (32)	42 (27)	0.006**
Myeloma	10 (26)	27 (23)	37 (23)	0.83**
Type of treatment, *n* (%)				
HDC with AHCT	13 (33)	68 (57)	81 (51)	0.02**
Median duration of neutropenia (days) [Min–Max]	7.5 [2; 29]	10 [3; 68]	9	0.12***
Microbial carriages, *n* (%)				
Bacterial	15 (38)	61 (51)	76 (48)	0.19**
Fungal	1 (3)	11 (9)	12 (8)	0.06**
Infection, *n* (%)				
Bacterial	5 (13)	43 (36)	48 (30)	0.008**
Including bacteraemia	3 (7)	31 (26)	34 (22)	0.02**
Fungal	1 (3)	9 (8)	10 (6)	0.45**
Death, *n* (%)	—	3 (3)	3 (2)	0.99**
Biological results (median [IQR])				
CLU (μg/mL)				
Inclusion	276.3 [230; 320]	238.6 [182; 280]		0.003***
Day of fever	—	204.5 [150; 252]		
PTX3 (ng/mL)				
Inclusion	0.38 [0.18; 0.63]	0.35 [0.16; 0.61]		0.76***
Day of fever	—	0.8 [0.43; 1.5]		

*Note*: The initial patient characteristics were retrieved from medical records and categorized based on their ‘febrile’ status. Categorical variables were compared using *χ* ^2^* or Fisher's** exact tests, while continuous variables were assessed using the Mann–Whitney test***. Bold values indicate significant of *p* < 0.05.

Abbreviations: AHCT, autologous haematopoietic cell transplantation; ALL, acute lymphoblastic leukaemia; AML, acute myeloid leukaemia; CLU, clusterin; HDC, high‐dose chemotherapy; IQR, interquartile range; *n*, number of events; PTX3, pentraxin 3.

Sixty‐two events (52%) were FN without sepsis (group 1), while 42 (35%) were a sepsis (group 2) and 14 (12%) were severe sepsis (8 events) or septic shock (6 events) (group 3) (Figure 1; Table S1). Eighteen FN events (16%; missing data [MD]: 7) were classified with a qSOFA score ≥2 (Figure 1; Table S2). Patients in groups 2 and 3 had more bacterial (*p* < 10^−4^) and fungal (*p* < 10^−4^) carriages than patients in group 1. As expected, they developed significantly more bacteraemia (*p* < 10^−4^) than patients from group 1. Patients with a qSOFA score ≥2 also had more fungal documentations (*p* < 10^−4^) and developed more fungal infection than those with a qSOFA score <2 (*p* = 0.02). Three patients died during FN. All these patients were included in group 3 and had a qSOFA score ≥2. Group 3 (*p* < 10^−4^) and a qSOFA score ≥2 (*p* = 0.004) were both associated with a higher risk of death.

Depending on the classification used, the proportion of ‘high‐risk’ events thus varied, from 11% considering group 3 to 16% for patients with qSOFA ≥2 (Figure 1). Eleven events were classified in both ‘high‐risk’ groups (group 3 + qSOFA ≥2), while discrepancies were found for eight events: two patients classified as group 3 (for severe sepsis and septic shock) did not have a qSOFA ≥2, and six patients with a qSOFA ≥2 did not have group 3 criteria (three SIRS without sepsis and three non‐severe sepsis). Among the three patients in group 1 with a qSOFA ≥2, two had hypotension associated with mild confusion (Glasgow score = 14) and one had neurological and respiratory signs considered to be side effects of oxycodone.

### Variations in PTX3 and CLU levels according to the severity of the FN episode

**FIGURE 2:**
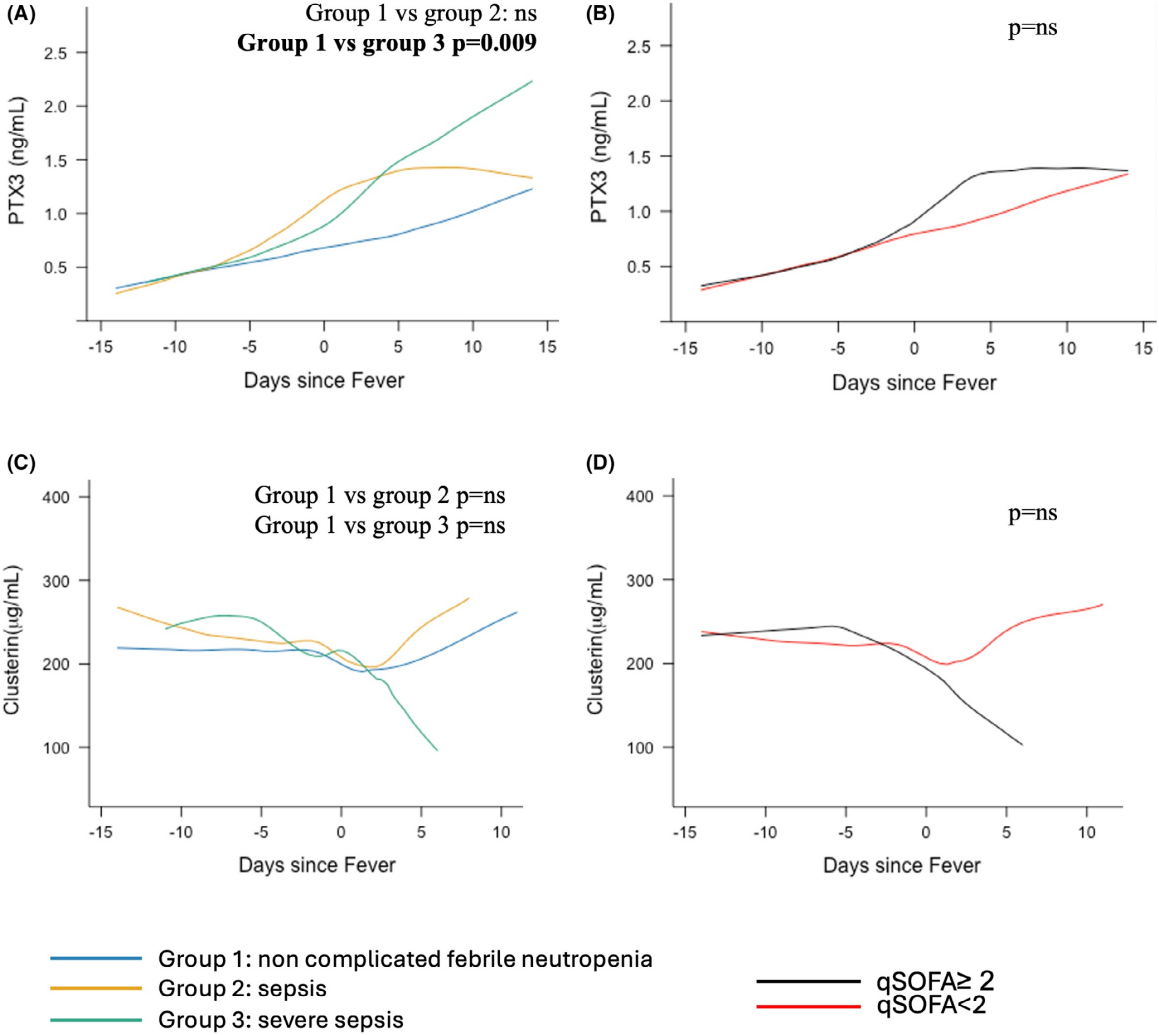
Kinetic variation of pentraxin 3 (PTX3) and clusterin (CLU) levels in serum from patients with febrile neutropenia. Day 0 indicates the first day of fever. (A) PTX3 levels (ng/mL) in patients with febrile neutropenia over time (in days) according to the severity of the febrile neutropenia. (B) PTX3 levels (ng/mL) in patient with febrile neutropenia over time (in days) according to Sequential Organ Failure Assessment (SOFA) score. (C) CLU levels (μg/mL) in patient with febrile neutropenia over time (in days) according to the severity of the febrile neutropenia. (D) CLU levels (ng/mL) in patient with febrile neutropenia over time (in days) according to SOFA score. All the results are obtained through a linear mixed model; fixed effects were time and time^2^.

At D0 (day of fever onset), serum PTX3 levels were higher in patients with severe sepsis than in patients with fever without sepsis (*p* = 0.02) (Table S1). Longitudinal analysis of all available samples showed significant variations in PTX3 levels over time (*p* = 0.01). Moreover, these variations differed significantly according to the sepsis severity groups studied, with a significantly higher elevation of PTX3 levels in group 3 than in group 1 (*p* = 0.009) (Figure 2A). CLU levels also varied over time (<10^−4^), but without significant differences between the three groups (Figure 2C). Longitudinal analysis of Interleukine 6 (IL‐6) and Tumor Necrosis Factor alpha (TNFα) levels showed the same trends as PTX3, with higher levels from D0 onwards in group 3 (respectively, *p* = 0.02; *p* = 0.008) (Figure S1A,E). No significant difference was found between groups for IL‐8 and CRP (Figure S1C,G). White blood cells (WBCs) did not appear to differ between groups (non‐significant) (not shown). PTX3 and pro‐inflammatory cytokine levels increased according to sepsis severity in patients with FN.

At D0, PTX3 (Figure 2B) and cytokines (IL‐6, Interleukine 8 (IL‐8), TNFα) (Figure S1B,D,F) showed no significant differences in either levels or trajectory model when classified according to qSOFA. The longitudinal trajectory model tended towards a steeper decrease in CLU level in patients with a qSOFA score ≥2; however, it was non‐significant (Figure 2D). CLU levels tended to decrease with increasing qSOFA scores in patients with FN, but none of the parameters investigated in our study were significantly different according to qSOFA score.

No association was found between CLU or PTX3 and mortality, but the number of cases was low (three patients).

### Variations in PTX3 and CLU levels according to type of infection

At D0, PTX3 levels were significantly higher in patients with an identified bacterial infection (0.98 ng/mL) than in patients without identified bacterial infection (0.67 ng/mL) (*p* < 0.05). No significant difference was found between patients with or without bacteraemia nor between patients with or without fungal infection. No significant difference was found in CLU level according to the type of infection.

Elevated PTX3 levels appeared to be associated with bacterial infection during FN.

### Correlation of CLU and PTX3 with inflammatory markers

**FIGURE 3:**
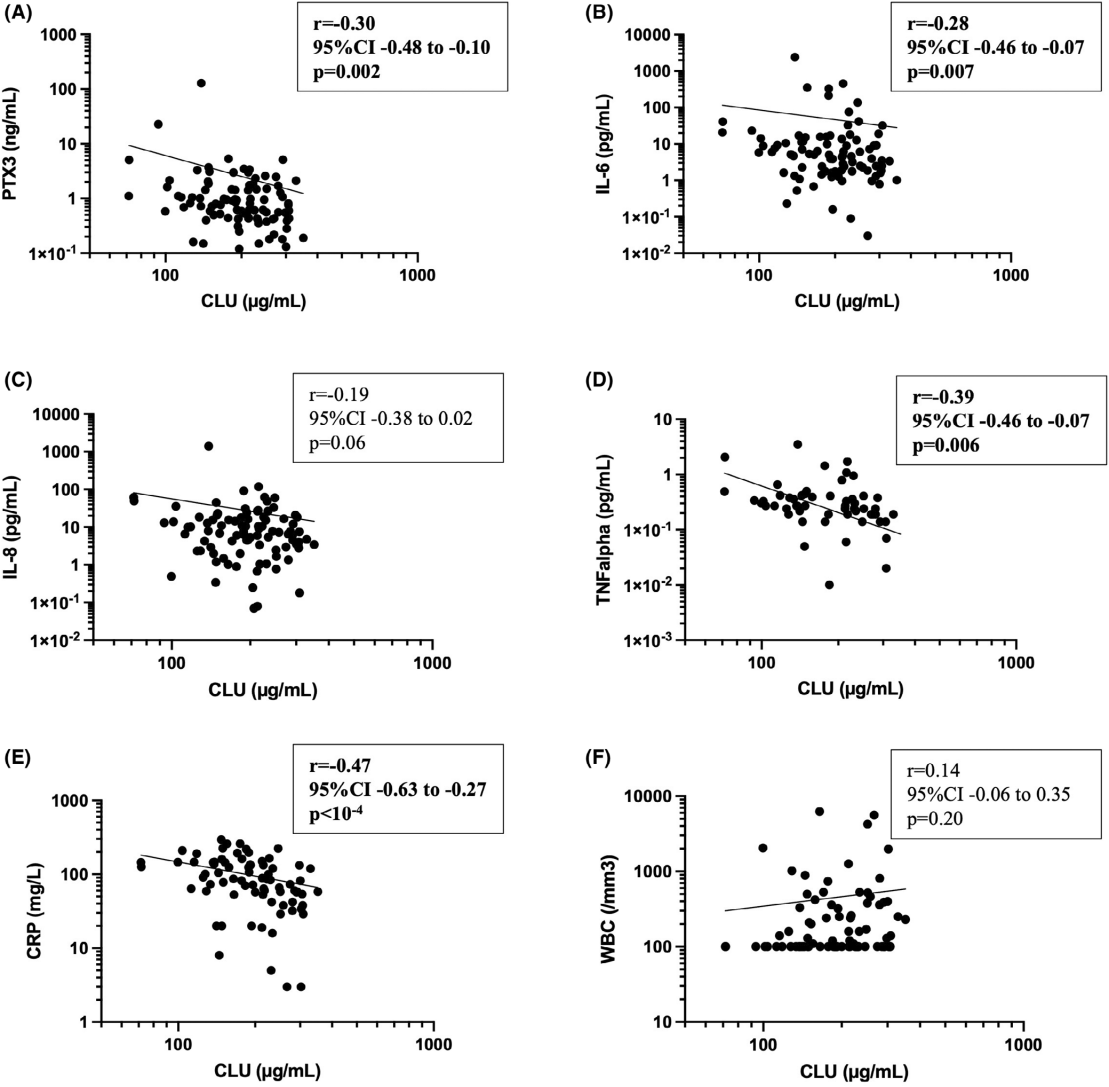
Correlation between clusterin (CLU) and pentraxin 3 (PTX3), IL‐6, IL‐8, TNFα, C‐reactive protein (CRP), serum and white blood cell (WBC) levels on the first day of fever (D0). Serum levels are represented on logarithmic scales for both axes. Lines depict non‐linear regressions. Correlations are assessed using nonparametric Spearman correlation tests, as the distributions are not Gaussian. ‘r’ represents Spearman's coefficient. (A) Correlation between CLU and PTX3 levels. (B) Correlation between CLU and IL‐6 levels. (C) Correlation between CLU and IL‐8 levels. (D) Correlation between CLU and TNFα levels. (E) Correlation between CLU and CRP levels. (F) Correlation between CLU and WBC levels.

Finally, we assessed the correlation between CLU, PTX3, and pro‐inflammatory cytokine levels. At D0, CLU levels were statistically negatively correlated to PTX3, IL‐6, TNFα and CRP levels (Figure 3), while PTX3 levels were positively correlated with IL‐6, IL‐8, TNFα and CRP levels (Figure S2). These observations confirm that the inflammatory response during severe infections in patients with FN is associated with an increase in serum PTX3 levels and a decrease in serum CLU levels.

## DISCUSSION

In this study, we evaluated variations of PTX3 and CLU levels in patients with FN after chemotherapy for haematological malignancies. PTX3 levels increased in patients experiencing severe sepsis, irrespective of qSOFA, were associated with bacterial infections, and correlated with pro‐inflammatory cytokines and CRP. CLU levels tend to decrease in patients with severe sepsis and qSOFA ≥2 and correlated inversely with pro‐inflammatory cytokines (IL‐6, TNFα) and inflammatory markers (CRP).

Patients included in our study had initial characteristics similar to those in recent studies, particularly regarding the type of disease.^1^, ^27^ Notably, the number of severe events and deaths attributable to infection was particularly low in our study in comparison to previous studies,^1^, ^4^ but nevertheless close to more recently published data.^27^ Severe infectious events have been steadily decreasing as supportive care in haematology has progressed, alongside improved prognosis due to better disease management and new therapies. With the enhancement of haematology‐related prognosis, it is becoming far less tolerable for deaths to be due to iatrogenesis. Therefore, studies evaluating early markers of severity are essential, as the time to initiate appropriate treatment is crucial in these pathologies.^1^ The parallel aim is to identify patients with lower risk of complications, even in populations usually considered at risk such as those included in our study. This approach could help reduce treatments, thereby improving quality of life and reducing adverse effects. Variations in CLU and PTX3 levels appeared to differ according to classification. Using the 2001 International Sepsis Definition, according to SIRS/Sepsis/Severe sepsis—Septic shock status,^23^ PTX3 is significantly higher in patients with severe sepsis or septic shock. With this classification, CLU decreased sharply but not significantly. In contrast, according to the current qSOFA classification,^24^ used in Sepsis‐3 definition proposed in 2016, CLU levels decreased more in patients with qSOFA ≥2, indicating a high risk of sepsis‐related mortality, while PTX3 seemed less marked with this classification. Thus, PTX3 and CLU could have distinct purposes in the assessment of FN. Historically, the Sepsis‐3 classification was proposed because the previous classification, which used the SIRS notion, was considered to be too sensitive and not specific enough regarding the infectious origin.^28^ However, in our study, CLU, which seems more closely associated with qSOFA, appears to be more closely linked to all‐cause severity, whereas PTX3 is associated with the 2001 classification and the presence of a confirmed bacterial infection.

PTX3 has been studied as a biomarker in numerous studies on sepsis or septic shock. Our study is the largest cohort to date evaluating PTX3 in FN,^14^, ^15^ and the only one assessing PTX3 according to qSOFA in this context. The role of PTX3 is well established in sepsis due to bacteria, with a predictive value for severe sepsis and mortality consistently demonstrated,^9^, ^10^, ^11^, ^29^ along with a strong correlation between PTX3 levels and SOFA score. Our results confirmed that elevated serum PTX3 levels were significantly associated with sepsis or severe sepsis/septic shock and with bacterial infection. However, we did not find an association between PTX3 levels and the qSOFA score, in contrast to studies conducted in non‐neutropenic populations; thus, PTX3 appears insufficient to assess the risk of mortality in FN. A potential hypothesis is that PTX3 is mainly synthesized by myeloid cells,^7^ including neutrophils,^8^, ^25^ whose numbers are reduced during FN. This reduction does not allow an increase in PTX3 production to a significant level in this context. Furthermore, the number of patients with a qSOFA score ≥2 is low in our study, which may fail to highlight a difference related to this score. None of the parameters studied were significantly different according to qSOFA score. Thus, we cannot conclude that PTX3 is a biomarker of high risk of mortality in patients with chemotherapy‐induced neutropenia, although it remains a strong marker of severe infections.

Our study is the first study assessing CLU in neutropenic patients. CLU has been described as associated with infections since its earliest descriptions. Low circulating CLU levels have been reported in bacterial infections (*Streptococcus pyogenes*, *Neisseria meningitidis*, *Streptococcus pneumoniae*, *Staphylococcus aureus*),^30^, ^31^ as well as in viral infections, such as dengue virus^32^ or coronavirus OC43^33^ infections. The mechanisms behind these observations remained unclear until recent studies highlighted the role of CLU in severe sepsis, its association with mortality and its potential use as a biomarker of severity.^21^, ^34^ CLU levels decrease during severe sepsis due to consumption, including through binding to extracellular histones released during severe inflammation.^21^ In our study, it was challenging to assess the role of CLU in evaluating mortality, due to the small number of events. However, CLU levels appeared to decrease more in patients with a high qSOFA, which is associated with a higher risk of mortality, although these results were not significant. This suggests that CLU may serve as a severity biomarker rather than as a specific biomarker of infection. In addition, CLU is ubiquitous in the human body^35^ and its synthesis should not be significantly altered by the decrease in haematopoietic cells, making it an interesting marker in haematology patients. However, a probably significant proportion of the extracellular histones released during severe infection comes from NETosis. Consequently, the amount of extracellular histones to be cleared during infection may be lower in neutropenic patients, who have fewer neutrophils compared to other patients with severe infections. This could potentially explain the smaller decrease in CLU levels observed in this cohort compared to previous studies,^21^ as its chaperone activity on histones and their scavenging would be less significant, even in severe patients. Further studies will be needed to assess the role of CLU in patients with neutropenia.

Few severe events were reported, and even less post‐infectious deaths, reflecting the improvement in supportive care in haematological malignancies. While this is obviously positive for patients, it reduced the power of our results, as these outcomes were not anticipated due to their absence in previous studies. Consequently, none of the markers evaluated were significantly affected by qSOFA, including pro‐inflammatory cytokines. CLU levels seemed to be most associated with qSOFA, but this association was not significant. Another challenge was compliance with the study schedule. Many patients could not be sampled at the strict D0 and D2 dates, as planned in the protocol, necessitating the use of trajectory analyses to study all enrolled patients. Additionally, we encountered changes in scoring system during our study. The Sepsis‐3 classification was proposed after our study began, which is why we proposed to study both 2001 and 2016 classifications. This classification has been questioned in recent years,^36^ particularly with regard to the capacity of the qSOFA as a rapid assessment tool for severity. Other so‐called ‘Early Warning Scores’, such as the NEWS (National Early Warning Score),^37^ could be used to identify the risk of mortality at an earlier stage. It appears to be as effective as qSOFA in assessing mortality risk,^38^ and further studies are needed to compare the added value of each.

PTX3 and CLU could therefore be valuable for the early assessment of patients with FN. PTX3 may be used to evaluate sepsis intensity and aetiology, while CLU appears to be a more appropriate biomarker for mortality risk in this context. This study should be extended to confirm this hypothesis and possibly develop a score adapted to neutropenic patients.

## AUTHOR CONTRIBUTIONS

Coralie Mallebranche: conceptualization, investigation, visualization, formal analysis, original draft preparation, review and editing. Carole Mosnier: conceptualization, investigation, original draft preparation, review and editing. Corentin Orvain: conceptualization, investigation, review and editing. Jérémie Riou: formal analysis, software, review and editing. Sylvain Thépot: conceptualization, review and editing. Pascale Pignon: investigation, review and editing. Simon Blanchard: investigation, review and editing. Nathalie Tortevoie: investigation, review and editing. Isabelle Pellier: conceptualization, review and editing. Mathilde Hunault‐Berger: conceptualization, review and editing. Pascale Jeannin: review and editing. Yves Delneste: conceptualization, review and editing. Aline Schmidt‐Tanguy: conceptualization, funding acquisition, investigation, visualization, original draft preparation, review and editing. Céline Beauvillain: conceptualization, formal analysis, funding acquisition, investigation, visualization, original draft preparation, review and editing.

## FUNDING INFORMATION

This study received a grant from Angers University Hospital. C.M. obtained a grant from the ‘Fondation pour la Recherche Médicale’.

## CONFLICT OF INTEREST STATEMENT

We have no conflicts of interest to disclose.

## ETHICS STATEMENT

This study was conducted in accordance with the Declaration of Helsinki and its later amendments and was approved by the national committee for the protection of individuals (CPP).

## PATIENT CONSENT STATEMENT

Written informed consent was obtained from all patients.

## Supporting information


Data S1.

Data S2.

Data S3.

## Data Availability

The data that support the findings of this study are available from the corresponding author upon reasonable request.
