# Supplementary material for: Clusterin and pentraxin 3 are markers of severity during febrile neutropenia in adults with haematological malignancies receiving intensive chemotherapy
Source: Br J Haematol. 2025 Jul 28;207(3):780–8. doi: 10.1111/bjh.70002 (PMC12436219; doi:10.1111/bjh.70002)
Supplement: Supplementary file 1 — Data S1. [file BJH-207-780-s002.doc]

**Supplementary Table 1: Patients characteristics according to ”sepsis severity group”**

The initial patient characteristics were collected from medical records and analysed according to the “sepsis severity” group. Categorical and continuous variables were compared by χ2* and by Kruskal-Wallis test**, respectively.


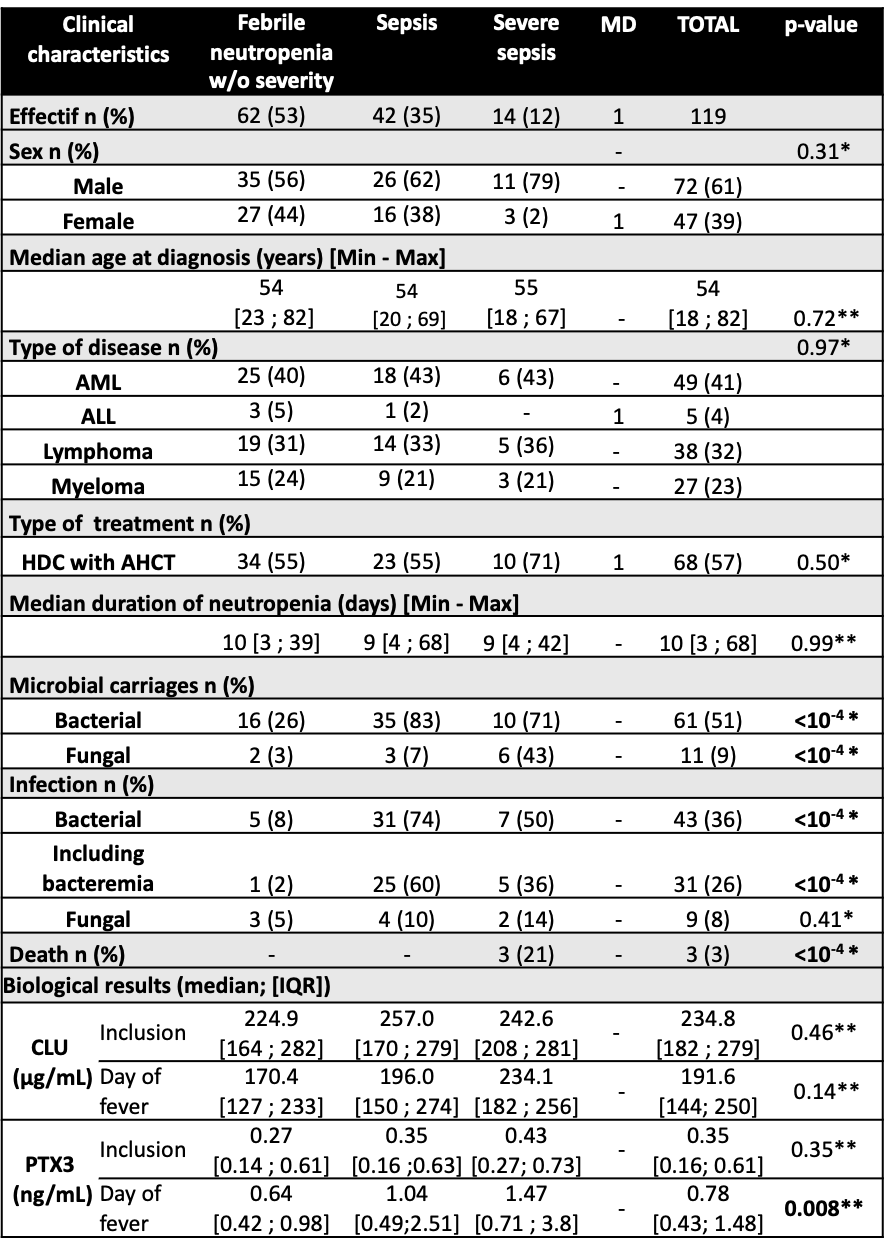


**Supplementary Figure 1 – Kinetic variations of IL-6, IL-8 , TNFα and CRP levels in serum and white blood cell numbers in febrile neutropenia patients. Day 0 indicates the first day of fever.** A. IL-6 levels (pg/mL) over time in patients with febrile neutropenia are depicted based on the severity of the febrile neutropenia. B. IL-6 levels (pg/mL) over time in patients with febrile neutropenia are displayed according to the SOFA score. C. IL-8 levels (pg/mL) over time in patients with febrile neutropenia are represented based on the severity of the febrile neutropenia. D. IL-8 levels (pg/mL) over time in patients with febrile neutropenia are presented based on the SOFA score. **E**. **TNFα** levels (pg/mL) over time in patients with febrile neutropenia are depicted based on the severity of the febrile neutropenia. F. **TNFα** levels (pg/mL) over time in patients with febrile neutropenia are displayed according to the SOFA score. G. CRP levels (mg/L) over time in patients with febrile neutropenia are presented based on the severity of the condition. H. CRP levels (mg/L) over time in patients with febrile neutropenia are displayed according to the SOFA score. All the results are obtained through a linear mixed model, fixed effects were time and time^2^.

Abbreviations: AHCT: autologous hematopoietic cell transplantation; ALL: acute lymphoblastic leukemia; AML: acute myeloid leukemia; ALL: acute lymphoblastic leukemia; CI95: confidence interval 95%; CLU: clusterin; n: number of events; HDC: high-dose chemotherapy; PTX3: pentraxin 3.

**Supplementary Table 2: Patients characteristics according to qSOFA group**

Initial characteristics of patients were collected from medical records and analysed according to the qSOFA group (SOFA < 2 or ≥ 2). Categorical and continuous variables were compared by χ2* or Fisher exact** tests where appropriate and by Mann-Whitney test***, respectively.


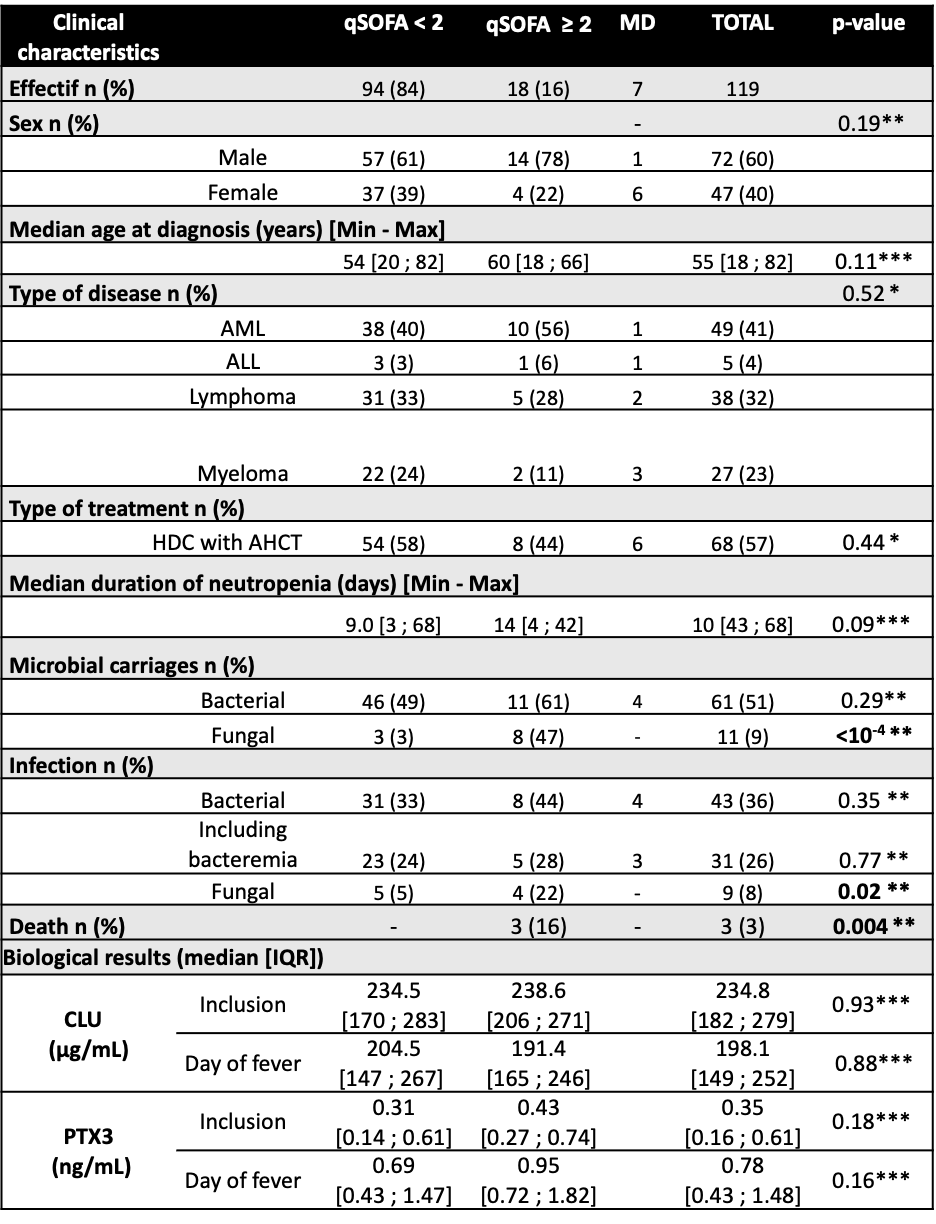


Abbreviations: AHCT: autologous hematopoietic cell transplantation; ALL: acute lymphoblastic leukemia; AML: acute myeloid leukemia; ALL: acute lymphoblastic leukemia; CLU: clusterin; n: number of events; HDC: high-dose chemotherapy; IQR: interquartile range; PTX3: pentraxin 3.

**Supplementary Figure 1 -** Kinetic variations of IL-6, IL-8 , TNFα and CRP levels in serum in febrile neutropenia patients.

**Supplementary Figure 2 -** Correlation between PTX3 and IL-6, IL-8, TNFα, CRP, levels in serum and White Blood Cell (WBC) levels on the first day of fever (D0).
