# Supplementary figures and images for: Clusterin and pentraxin 3 are markers of severity during febrile neutropenia in adults with haematological malignancies receiving intensive chemotherapy
Source: Br J Haematol. 2025 Jul 28;207(3):780–8. doi: 10.1111/bjh.70002 (PMC12436219; doi:10.1111/bjh.70002)

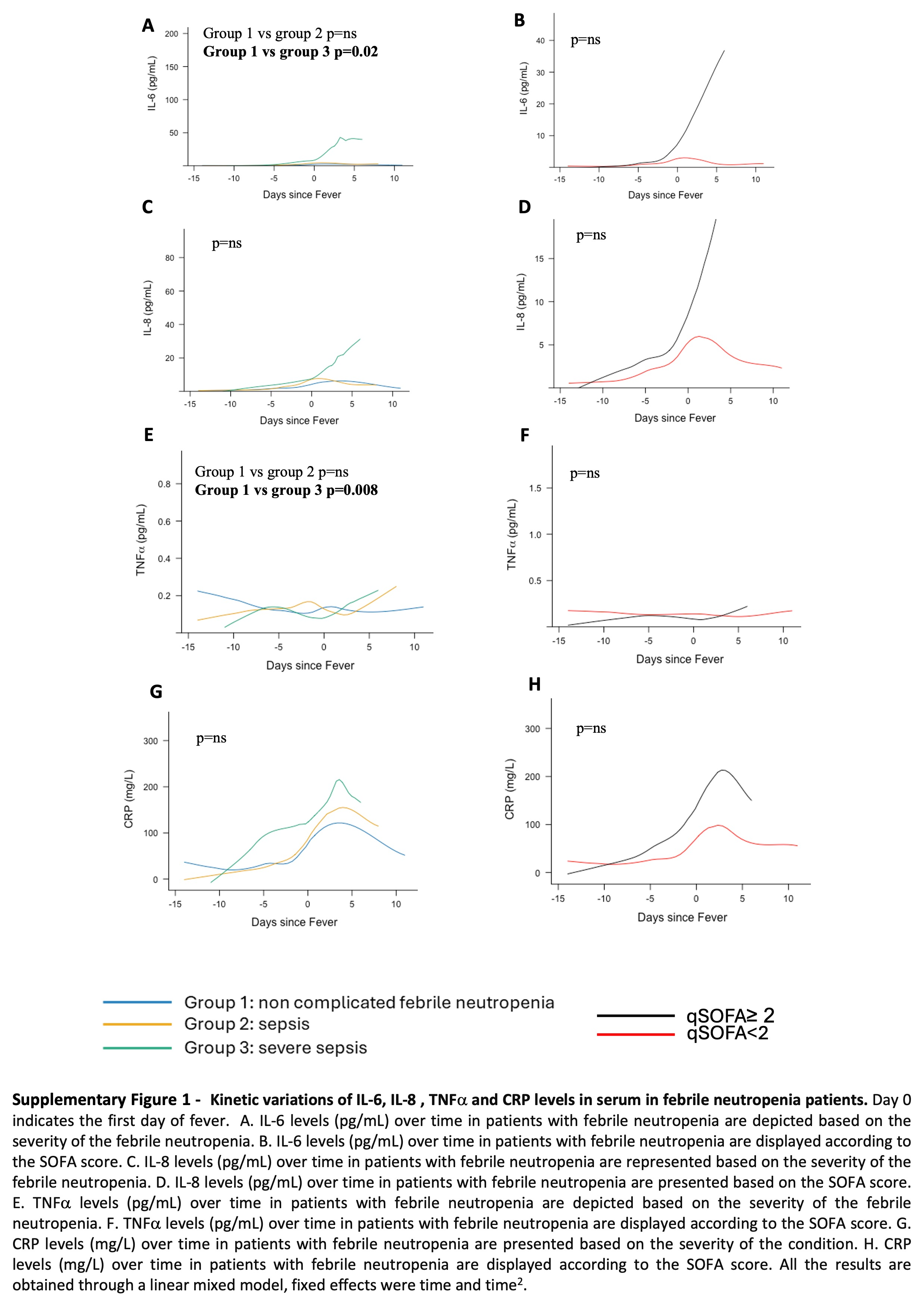

Supplement: Supplementary file 2 — Data S2. [file BJH-207-780-s001.tiff]

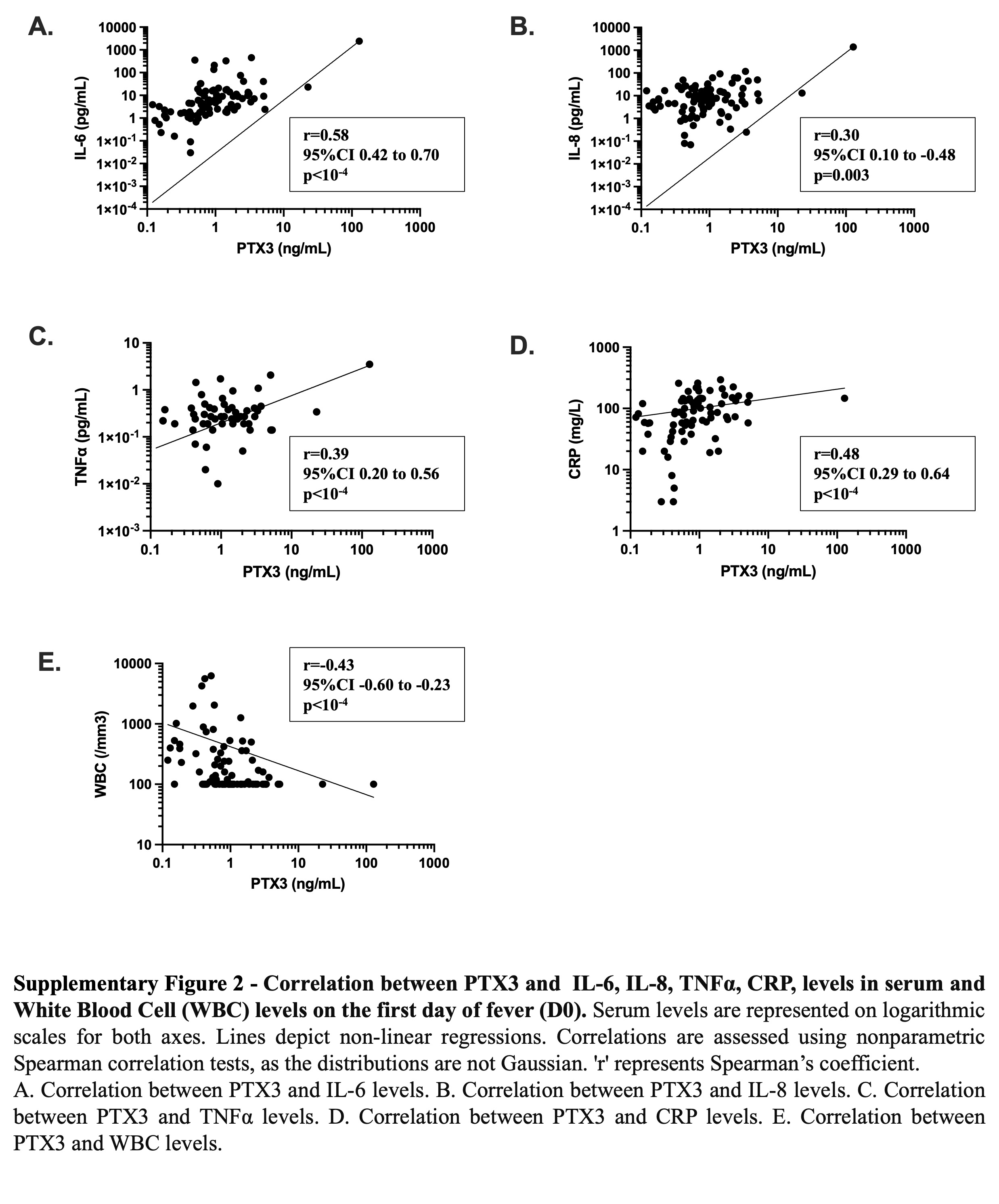

Supplement: Supplementary file 3 — Data S3. [file BJH-207-780-s003.tiff]
